# Can the Borg CR-10 scale for neck and low back discomfort predict future neck and low back pain among high-risk office workers?

**DOI:** 10.1007/s00420-022-01883-3

**Published:** 2022-06-01

**Authors:** Pooriput Waongenngarm, Allard J. van der Beek, Prawit Janwantanakul, Nipaporn Akkarakittichoke, Pieter Coenen

**Affiliations:** 1 Faculty of Health Science Technology, HRH Princess Chulabhorn College of Medical Science, Chulabhorn Royal Academy, Bangkok, Thailand; 2 Department of Physical Therapy, Faculty of Allied Health Sciences, Chulalongkorn University, Bangkok, Thailand; 3 Department of Public and Occupational Health, Amsterdam UMC, Vrije Universiteit Amsterdam, Amsterdam Public Health Research Institute, Van der Boechorststraat 7, 1081BT Amsterdam, The Netherlands

**Keywords:** Discomfort, Neck pain, Low back pain, Office workers, Predictive validity

## Abstract

**Purpose:**

Perceived discomfort could indicate an early sign of pain, for example, as a result of a biomechanical load on the musculoskeletal system. Assessing discomfort can, therefore, help to identify workers at increased risk of musculoskeletal disorders for targeted intervention development. We aimed: (1) to identify the optimal cut-off value of neck and low back discomfort among office workers and (2) to evaluate its predictive validity with future neck and low back pain, respectively.

**Methods:**

At baseline healthy participants (*n* = 100) completed questionnaires, including the Borg CR-10 discomfort scale (on a 0–10 scale), and were followed for six months, during which musculoskeletal pain was assessed monthly. Logistic regression analyses were performed to assess the associations of baseline discomfort with the onset of future neck or low back pain. Sensitivity, specificity, and the area under the receiver operating characteristics curve were estimated to identify the optimal discomfort cut-off value predicting future pain.

**Results:**

Borg CR-10 scores ≥ 3.5 for perceived neck and low back discomfort had acceptable sensitivity and specificity to predict future neck and low back pain, respectively. Perceived discomfort at baseline as a dichotomous measure (using the ≥ 3.5 cut-off) was a statistically significant predictor of future neck pain (OR = 10.33) and low back pain (OR = 11.81).

**Conclusion:**

We identified the optimal cut-off value of the Borg CR-10 discomfort scale to identify office workers at increased risk of developing neck and low back pain. These findings might benefit ergonomists, primary health care providers, and occupational health researchers in developing targeted interventions.

## Introduction

Neck and low back pain are a major health problem for many, and in particular for office workers. Neck pain is highly prevalent among office workers, as 46% of them reported neck pain annually (Ehsani et al. 2017) and 31% developed a new episode of neck pain every year (Areerak et al. 2018). Low back pain affects between 34 and 51% of office workers annually (Ayanniyi et al. 2010; Janwantanakul et al. 2008), while 14% developed a new onset of low back pain every year (Sitthipornvorakul et al. 2015). Neck and low back pain can result in significant physical and mental health issues, which can have an impact on work performance (Cote et al. 2008; Manchikanti 2000). Consequently, neck and low back pain put a large burden on individuals and the society as a whole.

Office work involves computer use, reading and participation in meetings, phone calls and presentations. Office work requires to sit for prolonged periods of time, which is often combined with repetitive (computer use) movements and sustained postures. Many individuals experience musculoskeletal discomfort during prolonged sitting, particularly in the neck and lower back area (Baker et al. 2018; Waongenngarm et al. 2015). However, also awkward postures and repetitive movements could play a role in the development of discomfort (Lis et al. 2007; Merino-Salazar et al. 2017). Discomfort during prolonged sitting is likely to be caused by increased muscle fatigue (Waongenngarm et al. 2016) and loads on passive structures (e.g. ligaments and intervertebral discs) (Mörl and Bradl 2013), and by a reduced intervertebral disc nutrition (Maroudas et al. 1975) and muscle blood flow (Credeur et al. 2019).

Signs of perceived bodily discomfort, such as from tension, fatigue, soreness, or tremors, can predict musculoskeletal pain (Hamberg-van Reenen et al. 2008). As such, perceived discomfort may indicate early signs of pain as a result of biomechanical load on the musculoskeletal system (Madeleine et al. 1997) and can therefore be used as an indicator of short-term effects of these loads. With insufficient recovery, these short-term effects could become recurrent or episodic and may eventually lead to musculoskeletal pain (van der Beek and Frings-Dresen 1998). Partly for this reason, in scientific studies, the effect of ergonomic interventions has been evaluated using perceived musculoskeletal discomfort as an early manifestation of musculoskeletal pain (Galinsky et al. 2007; McLean et al. 2001).

Several subjective measurement tools have been proposed to assess perceived discomfort, including numerical and graphic rating scales (Chanques et al. 2009; Iida et al. 2012; Shen and Parsons 1997). It has, however, been suggested that categorical scales are reliable and valid for the assessment of perceived discomfort, while numerical rating scales showed poor sensitivity and inconsistency (Shen and Parsons 1997). The Borg CR-10 scale is commonly used for the measurement of discomfort, as it has the advantages of being easy to use for laypeople with verbal descriptor of each numeric point, which has standard intervals and true zero points (Borg 1990). This tool has been found to be reliable and valid, i.e., showing high correlation with visual analogue scales (Capodaglio 2001).

Perceived discomfort could be one of the controllable risk factors of work-related musculoskeletal disorders, which can change from day to day, and can be alleviated by (ergonomic) interventions. Perceived discomfort may be a precursor of future neck and low back pain among office workers, which can easily be monitored and by which the short-term effects of (ergonomic) interventions can be assessed. An optimal cut-off value for a perceived discomfort could be useful to identify office workers at increased risk of developing neck and low back pain. This information can guide practitioners to develop preventive interventions for these workers. Moreover, the optimal perceived discomfort score can be used as a screening tool, which can help identify relevant participants to target interventions on (Moons et al. 2009). To date, however, no study has identified such an optimal cut-off value. Therefore, this study aimed to identify an optimal cut-off value of perceived neck and low back discomfort from the Borg CR-10 scale to predict office workers with future neck and low back pain, respectively. Additionally, using the identified optimal cut-off, we aimed to determine the predictive validity of neck and low back discomfort for future pain.

## Methods

### Participants and procedures

We analysed data from a prospective cohort study with 6-month follow-up (and monthly measurements) among a convenience sample of office workers from two large-scale Thai enterprises, i.e. a public transport operator and metropolitan waterworks authority. This study was conducted and reported according to the STROBE guidelines (Cuschieri 2019). Individuals were included if they were 18–55 years of age, worked full-time, had a body mass index (BMI) of 18.5–25 kg/m^2^ (depicting normal body weight according to WHO standards), had a seniority of at least 5 years in their current job, and were at high risk of developing neck and low back pain as assessed by a ≥ 2 score on the Neck Pain Risk Score for Office Workers (NROW) (Paksaichol et al. 2014) and a ≥ 53 score on the Back Pain Risk Score for Office Workers (BROW) (Janwantanakul et al. 2015). NROW consists of three questions regarding history of neck pain, chair adjustability, and perceived muscular tension, with summary scores ranging from 0 to 4. BROW consists of two questions regarding history of low back pain and psychological work demands, with summary scores ranging from 12 to 69.

Office workers who reported to have had neck or low back pain in the past 6 months, or a history of trauma or accidents affecting their spinal region, or surgery to either the spinal, intra-abdominal, or femoral region in the past 12 months were excluded. We also excluded workers who had been diagnosed with congenital anomaly of the spine, infections of the spine or discs, spondylolisthesis, ankylosing spondylitis, rheumatoid arthritis, spondylosis, spinal tumor, osteoporosis, or systemic lupus erythematosus. Workers who were pregnant or had planned to become pregnant in the following 12 months were also excluded. Eligible participants were screened on aforementioned criteria with a short screening questionnaire.

Eligible participants were informed about the goals and details of the study and were asked to give their informed consent after agreeing to participate. Participants completed questionnaires at baseline, which included the Borg CR-10 scale of neck and low back discomfort as well as additional personal and work-related characteristics. Participants were subsequently asked to complete diaries to assess any incidence, and if so, the intensity of neck and low back pain. Participants were asked to complete such a diary every month over a 6-month period. The study has been approved by the Chulalongkorn University Human Ethics Committee (COA No.148/2562).

### Baseline questionnaires

We used the Borg CR-10 scale to assess perceived neck and low back discomfort (Borg 1990), with body regions being defined with a diagram from the modified Nordic questionnaire (Kuorinka et al. 1987). Participants were asked about the intensity of discomfort in the past year on a 0–10 scale (with 0 being no discomfort and 10 extreme discomfort). Discomfort is considered a precursor of pain. As such discomfort can be a transient phenomenon resulting in pain, but not all pain can be attributed to discomfort (Ashkenazy and DeKeyser Ganz 2019). The definition of perceived discomfort used in this study is manifested in forms such as muscle fatigue, soreness, perceived tension, or tremors (de Looze et al. 2003).

In addition, the following personal and work-related characteristics were self-reported. Individual factors included age, gender, marital status, education level, smoking habits, frequency of participation in regular exercise/sport, and the number of driving hours per day. Work-related factors included current job, years of work experience, working hours, computer use (in hours/day), working postures, and the occurrence of various other work activities and rest breaks. The questionnaire also asked participants to rate (yes or no) their work environment regarding conditions (the appropriateness of ambient temperature, noise level, light intensity, and air circulation) and ergonomic configuration (whether the desk height was suitable for them, they used a height adjustable chair, and the top of the computer screen was positioned at a level horizontal with their eyes).

Psychosocial work characteristics were measured using the Thai version of the Job Content Questionnaire (Phakthongsuk 2009). The questionnaire comprises of 54 items regarding: psychological demands (12 items), decision latitude (11 items), social support (8 items), physical demands (6 items), job security (5 items), and hazards at work (12 items). Each item consisted of four Likert-type response options ranging from 1 (strongly disagree) to 4 (strongly agree). In each of the subscales, summary scores were calculated.

### Follow-up outcome measure

The area of potential neck and low back pain was determined with a chart of the body from the standardized Nordic questionnaire (Kuorinka et al. 1987). Furthermore, participants were asked to answer ‘yes’ or’no’ to the question “Have you experienced any neck or low back pain lasting > 24 h during the past month?”. In case of a ‘Yes’, questions regarding pain intensity were asked using a visual analogue scale. Participants were identified as having a new onset of neck and low back pain if they reported a pain intensity of > 30 out of 100 mm on a visual analogue scale (Sihawong et al. 2014; Sitthipornvorakul et al. 2020). Participants were followed until they completed all six-monthly questions or withdrew from the study.

### Statistical analysis

Means (with standard deviation) or proportions were used to describe participants’ characteristics. To maintain the statistical power of the database, a “hot-deck imputation” method was used to manage the missing data. According to this method, a participant was randomly selected from the total sample of participants with complete data, and the observed value for that participant was imputed for the participant for whom information was missing. This method was repeated for each missing value, until a complete dataset was obtained.

The 6-month incidence of neck and low back pain was estimated, while further follow-up data of those initially identified as case were not used further analysed. Baseline neck and low back discomfort were used as independent variable in two separate models with the incidence of either neck or low back pain during the 6-month follow-up period as dependent variable. Receiver operating characteristic (ROC) curves were plotted and the areas under the ROC curves (AUC) were estimated to assess the discriminatory ability of the Borg CR-10 scale of neck and low back discomfort to predict future pain. Sensitivity, specificity, positive predictive value (PPV), and negative predictive value (NPV) were calculated for various cut-off values of the Borg CR-10 scale, i.e. for 0.5, 1.5, 2.5, 3.5, 4.5, 5.5, 6.5, and 7.5. The Borg CR-10 score with the highest combined sensitivity and specificity was identified as the optimal cut-off value (Youden 1950). A perfect cut-off value (with 100% sensitivity and specificity) would have an AUC of 1.0. An AUC of 0.5 to 0.7 indicates no discriminatory ability above chance, 0.7 ≤ AUC < 0.8 indicates acceptable discriminatory ability, 0.8 ≤ AUC < 0.9 indicates excellent discriminatory ability, and an AUC ≥ 0.9 indicates outstanding discriminatory ability (Hosmer Jr et al. 2013).

Univariate analyses were conducted to determine the association between aforementioned personal and work-related characteristics and future neck or low back pain. Factors that predicted the outcome (with a *p* value ≤ 0.2) in univariate analyses were used in the multivariate analyses. Multivariate logistic regression analyses were performed to assess the associations between the perceived discomfort score at baseline and future neck and low back pain, respectively, adjusting for relevant confounders. This analysis was done for continuous Borg CR10 scores and for the dichotomous operationalisation of discomfort (using the optimal cut-off value of discomfort from the ROC curve analyses). Adjusted ORs and 95% confidence intervals were presented. All analyses were performed with SPSS for Windows Version 23.0 (SPSS Inc, Chicago, IL).

## Results

This study ran from June 2019 to December 2019. A total of 800 office workers received an invitation to participate in the current study, of whom 301 responded (initial response rate 38%). Of these, 201 did not meet the inclusion criteria and 100 were eligible; all of them agreed to participate in the study. There was no drop-out during the 6-month follow-up period and the percentage of missing data, for which we did imputation, was 0.06%. The study sample comprised mainly females (79%) and the average (standard deviation; SD) age was 34.5 (5.3) years. Most participants (95%) completed at least a bachelor’s degree. Table 1 shows the characteristics of the sample at baseline. During the 6-month follow-up period, 44 (44%) and 33 (33%) participants reported incidence of neck pain and low back pain, respectively.

**Table 1:** Characteristics of the study sample at baseline (*n* = 100)

Characteristics	*N* (%)	Mean (SD)	Max	Min
Personal characteristics				
Age (years)		34.5 (5.3)	46	24
Gender				
Male	21 (21)			
Female	79 (79)			
Marital status				
Single	64 (64)			
Married	35 (35)			
Divorced	1 (1)			
Education (%)				
Lower than Bachelor’s degree	5 (5)			
Bachelor’s degree	53 (53)			
Higher than Bachelor’s degree	42 (42)			
Exercise in the past 12 months (%)				
Never	22 (22)			
Occasionally	56 (56)			
Regularly	22 (22)			
Discomfort in the past 12 months				
Neck		4.1 (2.1)	9.0	0.0
Low back		3.3 (2.4)	9.0	0.0
Work-related characteristics				
Duration of employment (years)		9.1 (4.8)	21.0	5.0
Working hours (hours/day)		7.8 (0.8)	12.0	6.0
Working days (days/week)		5.0 (0.2)	7.0	5.0
Psychosocial characteristics				
Job control (11–44)		36.6 (4.3)	48.4	27.5
Psychological job demands (12–48)		33.2 (4.4)	45.0	22.0
Physical job demands (6–24)		14.1 (2.6)	22.0	7.0
Job security (5–20)		16.9 (1.1)	19.0	14.0
Social support (10–40)		32.9 (4.4)	40.0	23.5
Hazards at work (12–48)		17.0 (3.9)	28.0	12.0

*SD* standard deviation

In this study, three participants did not answer their age. Thus, three missing data were imputed. To examine the effect of missing data on our outcomes, results before and after the imputation procedure were compared, which did not show noticeable differences. Therefore, from this point forward only the results from the imputed dataset are reported.

**Fig. 1:**
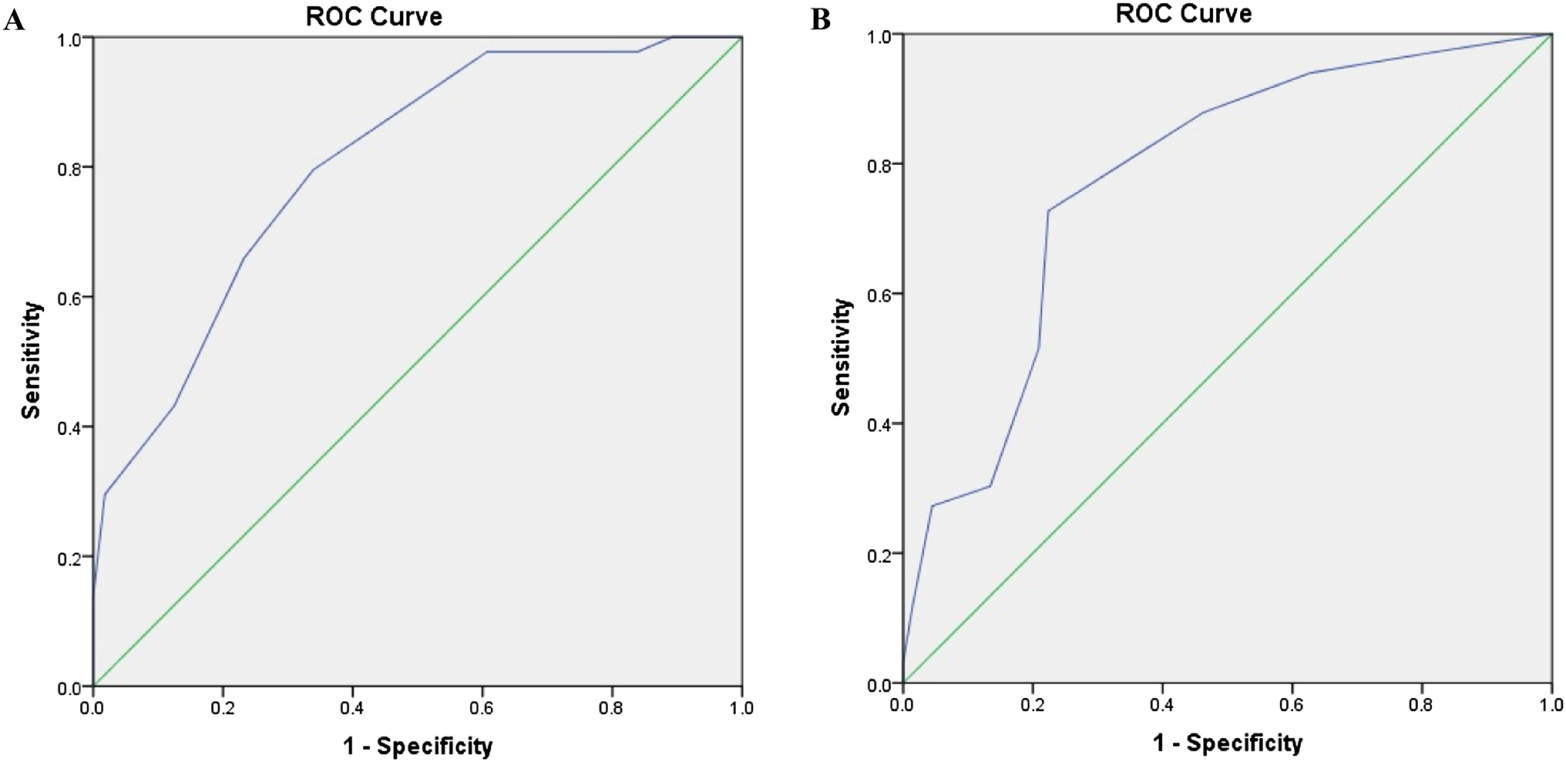
Receiver operating characteristic (ROC) curves for the association of discomfort and future pain within 6 months. **A** (left panel): for the neck region, and **B** (right panel): for the low back region

Table 2 presents sensitivity and specificity of all of the discomfort cut-off values for neck and low back pain. The most predictive cut-off value for perceived neck discomfort was ≥ 3.5 (sensitivity: 80%; specificity: 66%; PPV: 65%; and NPV: 80%). For this cut-off value, the AUC was 0.80 (95% CI 0.72–0.89) (Fig. 1A). The most predictive cut-off value for perceived low back discomfort was also ≥ 3.5 (sensitivity: 73%, specificity: 78%, PPV: 62%, and NPV: 85%), with an AUC of 0.77 (95% CI 0.68–0.87) (Fig. 1B).

**Table 2:** Sensitivity, specificity, positive predictive value (PPV), and negative predictive value (NPV) of each cut-off value of the Borg CR-10 score for neck and low back discomfort

Cut-off value	Sensitivity	Specificity	PPV	NPV
Neck discomfort				
≥ 0.5	100	10.7	46.8	100.0
≥ 1.5	97.7	16.1	47.8	89.9
≥ 2.5	97.7	39.3	55.8	95.6
≥ 3.5	79.5	66.1	64.8	80.4
≥ 4.5	65.9	76.8	69.1	74.1
≥ 5.5	43.2	87.5	73.1	66.2
≥ 6.5	29.5	98.2	92.8	63.9
≥ 7.5	13.6	100	100	59.6
Low back discomfort				
≥ 0.5	97	19.4	37.2	92.9
≥ 1.5	93.9	37.3	42.5	92.5
≥ 2.5	87.9	53.7	48.3	90.0
≥ 3.5	72.7	77.6	61.5	85.2
≥ 4.5	51.5	79.1	54.8	76.8
≥ 5.5	30.3	86.6	52.7	71.6
≥ 6.5	27.3	95.5	74.9	72.7
≥ 7.5	12.1	98.5	79.9	69.5

Table 3 shows univariate analyses for neck and low back pain incidence during the 6-month follow-up period. Regarding the univariate analyses for neck and low back pain, factors showing a *p* value ≤ 0.2 that were used for multivariate testing are shown in Table 4. Multivariate models that Borg CR-10 scores of perceived discomfort at baseline, both continuous and dichotomous (using the optimal cut-off value), were statistically significantly associated with pain incidence (Table 4). Perceived discomfort at baseline as a continuous measure was found to be a statistically significant predictor of future neck pain (OR_adj_ = 2.21; 95%CI = 1.45–3.39) and low back pain (OR_adj_ = 1.57; 95%CI = 1.20–2.06). Those with perceived discomfort score of ≥ 3.5 at baseline showed a statistically significantly increased risk of future neck pain (OR_adj_ = 10.33; 95%CI = 2.62–40.73) and low back pain (OR_adj_ = 11.81; 95%CI = 2.94–47.49).

**Table 3:** Univariate associations of all potential confounders for their association with neck and low back pain incidence during 6-month follow-up

Variable	Neck pain		Low back pain	
	OR (95% CI)	*p* value	OR (95% CI)	*p* value
Age (years)	1.02 (0.95–1.11)	0.498	1.07 (0.99–1.17)	0.088*
Gender				
Female	1.00		1.00	
Male	1.20 (0.46–3.16)	0.707	1.02 (0.37–2.83)	0.971
Marital status				
Single	1.00		1.00	
Married	1.82 (0.82–4.02)	0.139*	1.94 (0.86–4.40)	0.113*
Divorced	–	1.000	–	1.00
Education (%)				
Lower than Bachelor’s degree	1.00		1.00	
Bachelor’s degree	1.23 (0.62–2.46)	0.549	1.28 (0.62–2.65)	0.510
Higher than Bachelor’s degree	1.23 (0.83–1.84)	0.305	1.22 (0.80–1.86)	0.358
Exercise in the past 12 months (%)				
Never	1.00		1.00	
Occasionally	1.32 (0.72–2.40)	0.364	1.36 (0.72–2.58)	0.338
Regularly	1.31 (0.82–2.12)	0.262	1.39 (0.78–2.59)	0.259
Discomfort in the past 12 months				
Neck	1.92 (1.46–2.54)	0.000*		
Low back			1.57 (1.27–1.97)	0.000*
Work-related characteristics				
Duration of employment (years)	0.99 (0.91–1.07)	0.782	1.05 (0.96–1.14)	0.309
Working hours (hours/day)	1.33 (0.81–2.17)	0.257	0.97 (0.59–1.61)	0.919
Working days (days/week)	–	1.000	–	1.00
Psychosocial characteristics				
Job control (11–44)	1.09 (0.99–1.20)	0.084*	1.02 (0.92–1.12)	0.712
Psychological job demands (12–48)	1.13 (1.02–1.25)	0.016*	1.12 (1.01–1.24)	0.034*
Physical job demands (6–24)	1.13 (0.96–1.32)	0.145*	1.17 (0.98–1.39)	0.082*
Job security (5–20)	1.27 (0.87–1.86)	0.218	0.99 (0.68–1.47)	0.992
Social support (10–40)	1.05 (0.96–1.15)	0.304	0.99 (0.90–1.09)	0.896
Hazards at work (12–48)	1.08 (0.98–1.20)	0.124*	1.09 (0.98–1.21)	0.109*

**Table 4:** Logistic regression for the association between the Borg-CR10 scale for discomfort at baseline and neck and low back pain incidence during 6-month follow-up

Variable		Unadjusted		Adjusted	
		OR (95% CI)	*p* value	OR (95% CI)	*p* value
Neck discomfort at baseline (*n* = 44 developed neck pain (44%))	Continuous (Mean ± SD = 4.1 ± 2.1; Min = 0.0; Max = 9.0)	1.92 (1.46–2.54)	< 0.001	2.21 (1.45–3.39)^a^	< 0.001
Low back discomfort at baseline (*n* = 33 developed back pain (33%))	Continuous (Mean ± SD = 3.3 ± 2.4; Min = 0.0; Max = 9.0)	1.57 (1.27–1.97)	< 0.001	1.57 (1.20–2.06)^b^	0.001
Neck discomfort at baseline (*n* = 44 developed neck pain (44%))	< 3.5 (reference)	1.00		1.00	
	≥ 3.5	7.57 (3.02–18.96)	< 0.001	10.33 (2.62–40.73)^a^	0.001
Low back discomfort at baseline (*n* = 33 developed back pain (33%))	< 3.5 (reference)	1.00		1.00	
	≥ 3.5	9.24 (3.55–24.08)	< 0.001	11.81 (2.94–47.49)^b^	0.001

Odd ratios (OR) and 95% confidence intervals (95% CI) are shown (*N* = 100)

^a^Adjusted for marital status, driving, using computer more than 4 h, seat height, noise, temperature, air flow, rest breaks, monitor distance, keyboard level, job control, psychological job demands, physical job demands, and hazard at work

^b^Adjusted for age, marital status, driving, over-head activity, frequent neck flexion during work, seat height, noise, temperature, rest breaks, monitor distance, psychological job demands, physical job demands, and hazard at work

## Discussion

In this study we aimed to identify the optimal cut-off value of perceived neck and low back discomfort to predict future neck and low back pain and to evaluate the predictive validity of perceived neck and low back discomfort using the Borg CR-10 scale. Office workers without neck or low back pain in the previous 6 months but at risk of developing neck or low back pain were selected, to ensure that participants most in need of targeted interventions were represented. Office workers often sit for prolonged periods of time in front of a computer screen, which has been found to be associated with an increased level of musculoskeletal discomfort (Waongenngarm et al. 2020). Perceived discomfort can fluctuate, and increase and decrease from day to day. Perceived discomfort may therefore be an early sign for office workers to assess whether they will be at risk to develop future neck and low back pain.

We found optimal cut-off values for perceived discomfort to be 3.5 on the Borg CR-10 scale for both neck and low back discomfort, which when applied, provided an excellent ability to predict future incidence of neck and low back pain in office workers. The Borg CR-10 scale for perceived discomfort is therefore feasible as it can be carried out in a short period of time (less than 1 min, because it comprises only one question). This measure may be suitable for application in primary or occupational health care and workplace ergonomics settings, where full clinical examinations are not feasible due to limited resources. Also, the scale can be used in laboratory studies or other studies of relatively short duration, where there is not enough time for pain to develop. The cut-off values provided in the current study can be used in these contexts.

In this study we found that the 6-month incidence of neck and low back pain in office workers was 44% and 33%, respectively. Sitthipornvorakul et al. (2020), using the same incidence definition, found the incidence of neck pain in office workers to be 34%. Lapointe et al. (2009) reported 6-month incidences among neck and low back pain in office workers of 18% and 14%, respectively. Discrepancy between our and the latter study may be due to differences in case definitions and inclusion criteria. Lapointe et al. (2009) defined musculoskeletal pain by pain, ache, or discomfort with functional limitation at work, at home, or during leisure-time activities in the last six months. In our study, participants were identified as cases if they had pain lasting more than one day, with an intensity > 30 on a 100-mm visual analogue scale. Moreover, in the study by Lapointe et al. (2009) participants were not at particular risk of neck or low back pain, which was the case in our study.

In this study, a cut-off value of 3.5 showed to have the maximum sum of sensitivity and specificity for both the neck and low back region. For the neck, sensitivity, i.e. the ability of discomfort scores to identify high-risk workers when present, was 80%. As a result, the false-negative rate was 20%, which would mean that only 20% of high-risk office workers will falsely be identified as not being at high risk. With a cut-off value of 3.5, specificity, i.e. the ability of discomfort scores to identify low-risk workers when present, was 66%. As a result, the false-positive rate was 34%, which would mean that 34% of low-risk office workers will falsely be identified as being at high risk. For low back pain, the sensitivity was 73% and the specificity was 78%. A high false-positive rate would cost valuable resources as it would falsely identify workers that would not have benefited from any preventive intervention provided to them. However, participants in this study were at increased risk of developing neck and low back pain. As a result of this, the risks of false-negative should be weighed against the potential benefits of identifying a worker who is not at risk but receives an intervention (false-positive). A cut-off value with high sensitivity (low false-negative rate) would therefore be the preferred choice if the objective is to prevent as many workers as possible from developing neck and low back pain. In this study, the AUC was 0.80 for the neck and 0.78 for the low back, demonstrating that the Borg CR-10 scale for discomfort has acceptable to excellent discriminatory ability to identify workers likely and unlikely to develop future pain.

In clinical practice, predictive values are better applicable in decision making than sensitivity and specificity, because predictive values show the likelihood that the end-result is correct (Fritz and Wainner 2001). Our results showed that the predictive value of discomfort using a cut-off value of ≥ 3.5 was relatively low for the PPV and high for the NPV in both the neck and low back regions. For the neck, the PPV was 65%, which indicates that 65% of office workers with discomfort ≥ 3.5 were at risk of developing neck pain. The NPV was 80%, which means that 80% of office workers with discomfort < 3.5 were not at risk for developing neck pain. Similarly, the PPV and NPV for the low back were 62% and 85%, respectively. These findings suggest that the Borg CR-10 scale for discomfort is better suited to exclude office workers with a low risk of developing neck and low back pain in the future, than identifying workers with an increased risk of developing pain. However, it is important to note that while the PPV and NPV are important for interpreting the risk score, they strongly depend on the prevalence of the condition at study. For example, in samples with low disease prevalence, the PPV will be lower and the NPV will be higher (Fritz and Wainner 2001).

In this study we showed that perceived neck and low back discomfort at baseline (both continuous and dichotomous when using the ≥ 3.5 cut-off) were associated with future neck and low back pain, respectively. These findings suggest that neck and low back discomfort scores of ≥ 3.5 indeed predicted future neck and low back pain (with ORs of 10.33 and 11.81, respectively). This finding is in line with the study by Hamberg-van Reenen et al. (2008), in which it was shown that peak discomfort was a strong predictor of low back pain. Our findings are also in line with previous studies that have looked at muscular tension and physical demands (Huysmans et al. 2012; Wahlström et al. 2003). Perceived muscular tension has shown to be a strong predictor of future neck-shoulder complaints in symptom-free office workers (Huysmans et al. 2012). Wahlström et al. (2003) presented a model of computer work and musculoskeletal disorders in which both physical demands and mental stress at work could raise perceptions of muscular tension, which is, combined with discomfort, thought to be an early indicator of musculoskeletal disorders. Therefore, the optimal cut-off value of the Borg CR-10 discomfort scale (using the ≥ 3.5 cut-off) was a strong predictor of future neck and low back pain, and it may be utilized as a screening tool in healthcare settings and research.

The prospective design is a major strength of this study, which allowed us to assess the predictive ability of discomfort for future pain in office workers. In addition, the entire sample was successfully followed throughout the 6-month follow-up period. However, three limitations should be considered in the interpretation of our results. First, the external validity of this study is limited since we studied an office worker population with high-risk of neck and low back pain. This may affect the predictive validity of perceived discomfort to identify office workers with future neck and low back pain and generalizing our results to common office worker populations should be done with caution. Second, the study results should be restricted to office workers, and extrapolation of our findings to other occupational populations should be performed with caution. Finally, personal and work-related factors as well as neck pain and low back pain were self-reported. There is a risk of bias in all of these measures, which may have led to inaccuracy in our results. Future research should include objectively measured information from a physical assessment to improve data accuracy.

## Conclusion

Perceived discomfort, as measured with the Borg CR-10 scale, was a strong predictor for future neck and low back pain during a 6-month follow-up period. A cut-off value of ≥ 3.5 appeared to be optimal when predicting future pain, both in the neck and low back region. The Borg CR-10 scale for discomfort can feasibly be used by ergonomists and occupational health care providers. It could be valuable for early identification of office workers at high risk of developing neck and low back pain, for which interventions can then be developed. Furthermore, it might be useful in research of relatively short duration (e.g. laboratory studies), where there is not enough time for pain to develop. However, further validation and assessment of these methods in other populations of workers is suggested to increase external validity. Interventions that can decrease discomfort (e.g. workplace adjustments or rest break interventions) should be developed and evaluated for office workers with high-risk of the neck and low back pain for preventing future neck and low back pain.

## Data Availability

The data that support the findings of this study are available on request from the corresponding author. The data are not publicly available due to the ethical restrictions.
